# A Novel Robust *H*_∞_ Filter Based on Krein Space Theory in the SINS/CNS Attitude Reference System

**DOI:** 10.3390/s16030396

**Published:** 2016-03-18

**Authors:** Fei Yu, Chongyang Lv, Qianhui Dong

**Affiliations:** 1 College of Automation, Harbin Engineering University, Harbin 150001, China; yufei@hrbeu.edu.cn; 2 College of Information and Communication Engineering, Harbin Engineering University, Harbin 150001, China; dongqianhui@hrbeu.edu.cn

**Keywords:** uncertainty problem, SINS/CNS integrated system, missing measurements, Krein space theory, robust *H*_∞_ filter

## Abstract

Owing to their numerous merits, such as compact, autonomous and independence, the strapdown inertial navigation system (SINS) and celestial navigation system (CNS) can be used in marine applications. What is more, due to the complementary navigation information obtained from two different kinds of sensors, the accuracy of the SINS/CNS integrated navigation system can be enhanced availably. Thus, the SINS/CNS system is widely used in the marine navigation field. However, the CNS is easily interfered with by the surroundings, which will lead to the output being discontinuous. Thus, the uncertainty problem caused by the lost measurement will reduce the system accuracy. In this paper, a robust $H_{\infty }$ filter based on the Krein space theory is proposed. The Krein space theory is introduced firstly, and then, the linear state and observation models of the SINS/CNS integrated navigation system are established reasonably. By taking the uncertainty problem into account, in this paper, a new robust $H_{\infty }$ filter is proposed to improve the robustness of the integrated system. At last, this new robust filter based on the Krein space theory is estimated by numerical simulations and actual experiments. Additionally, the simulation and experiment results and analysis show that the attitude errors can be reduced by utilizing the proposed robust filter effectively when the measurements are missing discontinuous. Compared to the traditional Kalman filter (KF) method, the accuracy of the SINS/CNS integrated system is improved, verifying the robustness and the availability of the proposed robust $H_{\infty }$ filter.

## 1. Introduction

In modern marine navigation, the strapdown inertial navigation system (SINS) is widely used due to its advantages of being more compact and autonomous, which can provide vehicle’s navigation information, including attitude, velocity and position [1,2,3]. However, in SINS systems, there are accumulated navigation errors caused by the inertial components inevitably. Additionally, this is a serious problem in long-term marine navigation. Thus, some other navigation systems, e.g., the Global Position System (GPS), the Doppler velocity log (DVL), the celestial navigation system (CNS), *etc*., are often integrated with it to improve the navigation accuracy of the whole system availably making use of the complementary navigation information obtained from different sensors [4,5,6,7,8].

In CNS, the accurate attitude is calculated based on the azimuth of a celestial body measured by the celestial sensor. Since the celestial body is used as the navigation information source, the CNS has numerous merits, such as high positioning and orienting accuracy, good stealthiness and independence. What is more, the navigation error is not accumulated over time. Therefore, the CNS is widely used to aid the SINS in astronautics, marine navigation and surveying fields, utilizing the SINS/CNS integrated navigation system. In [9], Xu and Fang proposed INS/CNS integration, using the INS error model and Kalman filter (KF) based on neural networks. Integrating INS with GPS and a star tracker was performed by [10] using a decentralized multisensor integration structure, in which the error compensation rate of integration was studied. Because of that traditional ground-based initial alignment methods cannot work well on the lunar surface, [8] proposed a new autonomous INS initial alignment method, which used star observations to help the INS estimate its attitude, gyroscopes drifts and accelerometer biases. Additionally, simulations proved the superiority of the new method.

However, the CNS easily interfered with by the surroundings, such as clouds, since stars are used as the beacon of the star sensor. For example, the number of stars, which is used for calculating the navigation information of the star sensor, is reduce under cloudy weather. Hence, the occluded star sensor will lead to the output being discontinuous [6,11,12]. Then, the system model will not be accurate under this condition. Furthermore, the uncertainty problem will be introduced into the integrated system, reducing the system accuracy significantly. Although the KF is the most commonly-used optimal estimation, it is hard to get the expected results when the system model is not accurate. Therefore, many methods are used to describe and compensate the uncertainty of the system [13,14,15,16]. In [14], a new robust Kalman filter was proposed that detects and bounds the influence of outliers in a discrete linear system, including those generated by thick-tailed noise distributions, such as impulsive noise. Taking the robust state estimation for uncertain descriptor systems into account, a robust filtering framework (RFF) was proposed to facilitate the robust filter design [16]. Hamza and Nebylov proposed a robust design of an INS/GNSS navigation system to solve the problem of state space models with non-Gaussian measurement noise based on parallel nonlinear filtering [13].

Although the robust Kalman filter based on the $H_{2}$ norm has a simple design form, it requests that the statistical characteristics of the system noise be already known, which is difficult to meet in practical applications. The robust $H_{\infty }$ filter has high stability, but its design form is complex, which is not suitable for actual applications, as well [15,17].

In recent years, due to its simple design, flexible structure and wide application, the Krein space theory has become a hot issue gradually [18,19,20]. Therefore, a robust $H_{\infty }$ filter for the SINS/CNS integrated navigation system is presented in this manuscript based on the Krein space theory. Taking the uncertainty problem into account, a robust $H_{\infty }$ filter in the Krein space frame is presented and derived. Even better, the novel filter not only achieves robustness against missing measurements using robust $H_{\infty }$ filtering, but also improves the system accuracy effectively due to the Krein space theory. Additionally, the results from simulations and experiments show that the presented robust filter is superior to the normal Kalman filter. The rest of this manuscript is organized as follows. The fundamentals of the Krein space theory and the linear error equations of the SINS/CNS integrated navigation system are introduced in Section 2. Additionally, the new robust filter is proposed in Section 3. Numerical simulations and experiments along with specific analysis are given in Section 4. Section 5 concludes this manuscript.

## 2. Linear Filter Based on the Krein Space Theory

In this section, some basic knowledge of the Krein space theory will be introduced in order to understand the theoretical derivation below easily. The system model of the SINS/CNS integrated system is established, as well, here.

### 2.1. Fundamentals of the Krein Space Theory

The Krein space is a non-classical functional space, which has attracted extensive attention. The kernel of the Krein space estimation theory is that the minimization of the quadratic cost function is translated into the Kalman filter problem just in the Krein space, rather than the random Hilbert space [19,20,21].

Now, consider the following Krein space state-space formal system:

(1) $$\left\{\begin{array}{c} x_{j+1}=A_{j}x_{j}+B_{j}u_{j}\\ y_{j+1}=C_{j+1}x_{j+1}+D_{j+1}v_{j+1} \end{array}\right.$$

with covariance matrix:

(2) $$\langle \left(\begin{array}{c} x_{0}\\ u_{j}\\ v_{j+1} \end{array}\right),\left(\begin{array}{c} x_{0}\\ u_{j}\\ v_{j+1} \end{array}\right)\rangle =\left(\begin{array}{ccc} P_{0} & 0 & 0\\ 0 & Q_{j} & S_{j}\\ 0 & S_{j} & R_{j+1} \end{array}\right)$$

wherein $x_{j+1}$ and $y_{j+1}$ are the state vector and the measurement vector at time j+1, respectively; $A_{j}$ and $C_{j+1}$ are specific known linear functions, the state-transition matrix and the measurement matrix; $B_{j}$ and $D_{j+1}$ are the coefficient matrices of the state noises; $u_{j}$ and $v_{j+1}$ are the state noise vector and observation noise vector with their autocorrelation and cross-correlation matrix of variance matrices $Q_{j}$, $R_{j+1}$ and $S_{j}$, respectively; $P_{0}$ is the initial state covariance matrix.

Thus, the quadratic cost function is chose as follows:

(3) $$J=x_{0}^{T}P_{0}^{-1}x_{0}+\sum_{j=0}^{N}u_{j}^{T}Q_{j}^{-1}u_{j}+\sum_{j=0}^{N}v_{j}^{T}R_{j}^{-1}v_{j}$$

We define the state transition matrix as:

(4) $$\phi_{j,k}\overset{\Delta }{=}A_{j-1}\cdots A_{k},j>k,\phi_{j,j}=I$$

and the response matrix as:

(5) $$h_{j,k}\overset{\Delta }{=}C_{j}A_{j-1}\cdots A_{k+1}B_{k}$$

Then, using:

(6) $$\bar{y}\overset{\Delta }{=}col\left\{y_{0},\cdots {\bar{y}}_{N}\right\}\bar{y}\overset{\Delta }{=}col\left\{y_{0},\cdots {\bar{y}}_{N}\right\}\bar{v}\overset{\Delta }{=}col\left\{v_{0},\cdots ,v_{N}\right\}$$

the Krein space state-space as Equation (1) satisfies this:

(7) $$\bar{y}=\bar{\Theta }x_{0}+\bar{\Upsilon }\bar{u}+\bar{\Omega }\bar{v}=\left(\begin{array}{ccc} \bar{\Theta } & \bar{\Upsilon } & \bar{\Omega } \end{array}\right)\left(\begin{array}{c} x_{0}\\ \bar{u}\\ \bar{v} \end{array}\right)$$

wherein:

(8) $$\Xi \overset{\Delta }{=}\langle \left(\begin{array}{c} x_{0}\\ \bar{u}\\ \bar{y} \end{array}\right),\left(\begin{array}{c} x_{0}\\ \bar{u}\\ \bar{y} \end{array}\right)\rangle =\left(\begin{array}{ccc} I & 0 & 0\\ 0 & I & 0\\ \bar{\Theta } & \bar{\Upsilon } & \bar{\Omega } \end{array}\right)\left(\begin{array}{ccc} P_{0} & 0 & 0\\ 0 & \bar{Q} & \bar{S}\\ 0 & {\bar{S}}^{T} & \bar{R} \end{array}\right){\left(\begin{array}{ccc} I & 0 & 0\\ 0 & I & 0\\ \bar{\Theta } & \bar{\Upsilon } & \bar{\Omega } \end{array}\right)}^{T}$$

and the symbols are defined as follows:

(9) $$\begin{array}{c} \bar{Q}=Q_{0}⊕\cdots ⊕Q_{N}\\ \bar{R}=R_{0}⊕\cdots ⊕R_{N}\\ \bar{S}=S_{0}⊕\cdots ⊕S_{N}\\ \bar{\Omega }=D_{0}⊕\cdots ⊕D_{N} \end{array}$$

(10) $$\bar{\Theta }=\left(\begin{array}{c} C_{0}\\ C_{1}\phi_{1,0}\\ \vdots \\ C_{N}\phi_{N,0} \end{array}\right),\bar{\Upsilon }=\left(\begin{array}{cccc} 0 & & & \\ h_{1,0} & 0 & & \\ h_{2,0} & h_{2,1} & 0 & \\ \vdots & \cdots & & \ddots \end{array}\right)$$

The projection error in Krein space to study is the following Gramian:

(11) $$\langle æ-k\bar{y},æ-k\bar{y}\rangle =\left(\begin{array}{cc} I & -k \end{array}\right)\Xi \left(\begin{array}{c} I\\ -k^{T} \end{array}\right)$$

where we suppose $æ=\mathrm{col}\left\{x_{0},\bar{u}\right\}$.

Unfortunately, the partial equivalence between Equations (3) and (11) cannot be found as easily as in [22,23]; thus, some more matrix algebra has to be used to translate Equation (3) into our desired format.

In [20], zero-valued polynomials were introduce to proof that the deterministic quadratic form as Equation (3) has the same stationary point to the error Gramian as Equation (11) in Krein space. On the basis of Theorem 1 and Lemma 2, it can be deduced easily. Therefore, it will not be elaborated any more in this paper.

As we all know, the KF method has a recursive form, which is simply and easily achieved. Therefore, the classical recursion form of KF is used in filters of the Krein space. Consider the discrete-time linear state-space model and its *a priori* knowledge, shown as Equations (1) and (2); the Riccati recursive steps are summarized as follows:

The estimated state vector:

(12) $$\;{\hat{x}}_{j+1}=A_{j}{\hat{x}}_{j}+K_{j}\left(y_{j}-B_{j}{\hat{x}}_{j}\right)$$

The filtering gain:

(13) $$K_{j}=\left(A_{j}P_{j}C_{j}^{T}+B_{j}S_{j}\right)R_{e,j}^{-1}$$

wherein:

(14) $$\;R_{e,j}=C_{j}P_{j}C_{j}^{T}+R_{j}$$

The estimated covariance matrix:

(15) $$P_{j+1}=A_{j}P_{j}A_{j}^{T}+B_{j}Q_{j}B_{j}^{T}-K_{j}R_{e,j}K_{j}^{T}$$

### 2.2. SINS/CNS Integrated System Model

**Figure 1:**
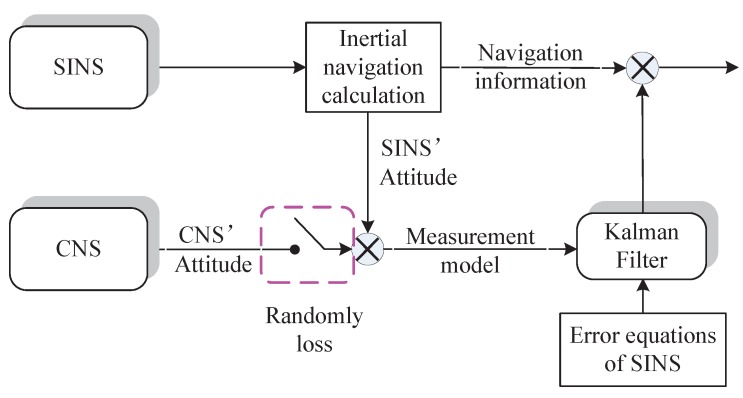
Schematic diagram of the SINS/CNS integrated system.

Although SINS has numerous advantages, the accumulated error caused by its inertial components limits its applications. The CNS can provide accurate attitude information based on the azimuth of the celestial body. Therefore, the SINS/CNS integrated navigation system is widely applied, since the navigation accuracy of the whole system can be enhanced significantly by using the complementary navigation information obtained from two different kinds of sensors. In the SINS/CNS integrated navigation system, the loosely-coupled scheme is used due to its simpleness and convenience. As we all know, the CNS is easy to interfere by the surroundings, and then, the CNS information will be invalid discontinuously. Taking this situation into account, the schematic diagram of the SINS/CNS integrated system is shown in Figure 1.

In this paper, we focus on marine navigation. As we all know, the vertical information (vertical acceleration, vertical velocity and vertical altitude) can be ignored for simplification reasonably and acceptably in surface navigation systems. Thus, only horizontal information is taken into account. Therefore, we choose the error equation of SINS as the state model, including horizontal velocity error equations, longitude and latitude error equations and attitude error equations.

(1) Velocity error equation:

(16) $$\begin{array}{cc} & \delta {\dot{V}}_{E}=\frac{V_{N}}{R_{n}}\tan L\delta V_{E}+\left(2\omega_{ie}\sin L+\frac{V_{E}}{R_{n}}\tan L\right)\delta V_{N}\\ & +\varphi_{U}f_{N}+\nabla_{E}+\left(2\omega_{ie}\cos LV_{N}+\frac{V_{E}V_{N}}{R_{n}}\sec^{2}L\right)\delta L \end{array}$$

(17) $$\begin{array}{cc} \delta {\dot{V}}_{N}= & -\left(2\omega_{ie}\sin L+\frac{2V_{E}}{R_{n}}\tan L\right)\delta V_{E}-\varphi_{U}f_{E}\\ & +\nabla_{N}-\left(2\omega_{ie}\cos LV_{E}+\frac{V_{E}^{2}}{R_{n}}\sec^{2}L\right)\delta L \end{array}$$

(2) Position error equation:

(18) $$\delta \dot{L}=\frac{\delta V_{N}}{R_{m}}$$

(19) $$\delta \dot{\lambda }=\frac{\delta V{}_{E}}{R_{n}}\sec L+\frac{V_{E}}{R_{n}}\tan L\sec L\delta L$$

(3) Attitude error equation

(20) $$\begin{array}{cc} {\dot{\varphi }}_{E}= & -\frac{\delta V_{N}}{R_{m}}+\left(\omega_{ie}\sin L+\frac{V_{E}\tan L}{R_{n}}\right)\varphi_{N}\\ & -\left(\omega_{ie}\cos L+\frac{V_{E}}{R_{n}}\right)\varphi_{U}+\varepsilon_{E} \end{array}$$

(21) $$\begin{array}{cc} {\dot{\varphi }}_{N}= & -\omega_{ie}\sin L\delta L+\frac{\delta V_{E}}{R_{n}}\\ & -\left(\omega_{ie}\sin L+\frac{V_{E}\tan L}{R_{n}}\right)\varphi_{E}-\frac{V_{N}}{R_{m}}\varphi_{U}+\varepsilon_{N} \end{array}$$

(22) $$\begin{array}{cc} {\dot{\varphi }}_{U}= & \left(\omega_{ie}\cos L+\frac{V_{E}}{R_{n}}\sec^{2}L\right)\delta L+\tan L\frac{\delta V_{E}}{R_{n}}\\ & +\left(\omega_{ie}\cos L+\frac{V_{E}}{R_{n}}\right)\varphi_{E}+\frac{V_{N}}{R_{m}}\varphi_{N}+\varepsilon_{U} \end{array}$$

wherein $V_{E}$ and $V_{N}$ are the east and north velocities and $\delta V_{E}$ and $\delta V_{N}$ are corresponding velocity errors; *L* and *λ* are the local latitude and longitude; while $R_{m}$ and $R_{n}$ are the Earth’s radii of the meridian circle and the prime vertical circle; $f_{E}$ and $f_{N}$ are the measured specific force by the east and north accelerometers; $\varphi_{E},\varphi_{N},\varphi_{U}$ are the attitude angle errors; $\varepsilon_{x}$, $\varepsilon_{y}$ and $\varepsilon_{z}$ are the gyro drifts of the *x*-, *y*- and *z*-axes; $\nabla_{x}$ and $\nabla_{y}$ are the accelerator biases of the *x*- and *y*-axes. Additionally, we also know this:

(23) $$\;\left(\begin{array}{c} \varepsilon_{E}\\ \varepsilon_{N}\\ \varepsilon_{U} \end{array}\right)=C_{b}^{n}\left(\begin{array}{c} \varepsilon_{x}\\ \varepsilon_{y}\\ \varepsilon_{z} \end{array}\right)$$

wherein $C_{b}^{n}$ is the transformation matrix from the vehicle’s body coordinate system (*b*) to the navigation coordinate system (*n*), the size of which is 3×3. A similar transform exists between $\nabla_{E},\nabla_{N}$ and $\nabla_{x},\nabla_{y}$.

From the above, the state vector x of the SINS/CNS integrated system is defined as:

(24) $$x(t)={\left[\begin{array}{cccccccccccc} \delta L & \delta \lambda & \delta V_{E} & \delta V_{N} & \varphi_{E} & \varphi_{N} & \varphi_{U} & \varepsilon_{x} & \varepsilon_{y} & \varepsilon_{z} & \nabla_{x} & \nabla_{y} \end{array}\right]}^{T}$$

The measurement is the attitude error between the calculated attitude of the SINS and the CNS. Therefore, the observation equation can be described as:

(25) $$z\left(t\right)=\left[\begin{array}{c} \varphi_{E}\\ \varphi_{N}\\ \varphi_{U} \end{array}\right]=H\left(t\right)x\left(t\right)+v\left(t\right)$$

where z(t) denotes the measurement, while v(t) is the measurement noise. The measurement matrix:

(26) $$H\left(t\right)=\left[\begin{array}{ccc} 0_{3\times 4} & I_{3\times 3} & 0_{3\times 5} \end{array}\right]$$

## 3. Robust $H_{\infty }$ Filter Based on Krein Space Theory

In this section, we formulate the Krein space filter recursions for the $H_{\infty }$ filter problem. Taking the missing measurements into account, a novel robust $H_{\infty }$ filter based on the Krein space theory is proposed.

Considered the following discrete-time linear state-space system:

(27) $$\left\{\begin{array}{c} x_{j+1}=\left(A_{j}+\Delta A_{j}\right)x_{j}+B_{j}u_{j}\\ y_{j+1}=\left(C_{j+1}+\Delta C_{j+1}\right)x_{j+1}+v_{j+1} \end{array}\right.$$

wherein $x_{j}$ and $y_{j+1}$ are the state vector and measurement vector, $u_{j}$ and $v_{j}$ are unknown system noises and $\Delta A_{j}$ and $C_{j}$ are uncertainty parameters. Generally, we assume that:

(28) $$\left(\begin{array}{c} \Delta A_{j}\\ \Delta C_{j} \end{array}\right)=\left(\begin{array}{c} F_{j}\\ D_{j} \end{array}\right)\Delta_{j}E_{j}$$

wherein $A_{j},B_{j},C_{j},D_{j},E_{j}$ and $F_{j}$ are already known matrices and $\Delta_{j}$ is a unknown matrix with:

(29) $$\Delta_{j}^{T}\Delta_{j}\leq I$$

Given $\xi_{j}=\Delta_{j}E_{j}x_{j}$, $s_{j}=E_{j}x_{j}$, the system uncertainty can be indicated by the following constraint condition:

(30) $$\;{∥\xi_{j}∥}^{2}\leq {∥s_{j}∥}^{2},j=0,1,\ldots ,N$$

where $∥\cdot ∥$ denotes the standard $H_{2}$ norm.

In a general way, the estimated vector is the linear combination of the state vector. That means:

(31) $$\;z_{j}=L_{j}x_{j}$$

wherein $L_{j}$ is a known coefficient matrix.

Defining that ${\hat{z}}_{j}$ is the estimation of $z_{j}$ on the basis of the given observations $\left\{y_{j}\right\}$, the estimation error is:

(32) $$\;e_{j}=z_{j}-{\hat{z}}_{j}$$

Thus, the estimation problem of the $H_{\infty }$ filter under the level parameter *γ* can be translated into solving the optimal solution of Equation (27):

(33) $$\;J={∥T∥}_{\infty }^{2}=\underset{x_{0},u_{j},v_{j}}{\sup}\frac{\sum_{j=0}^{N}e_{j}^{T}e_{j}}{{x_{0}}^{T}{P_{0}}^{-1}x_{0}+\sum_{j=0}^{N}u_{j}^{T}Q_{j}^{-1}u_{j}+\sum_{j=0}^{N}v_{j}^{T}v_{j}}\leq \gamma^{2}$$

From the above, we can see that the $H_{\infty }$ filter should be designed to ensure that the energy of estimation error $e_{k}$ is *γ* times less than the one of system noises. If Equation (33) can be guaranteed, the estimation error will be very small authentically. Substituting, the constraint of uncertain parameters shown as Equation (30) into Equation (33), we will obtain the robust $H_{\infty }$ filter problem:

(34) $$\;\underset{J_{\infty }\left(x_{0},u,v\right)}{\underset{︸}{{x_{0}}^{T}{P_{0}}^{-1}x_{0}+\sum_{j=0}^{N}u_{j}^{T}Q_{j}^{-1}u_{j}+\sum_{j=0}^{N}v_{j}^{T}v_{j}+\sum_{j=0}^{N}{∥\xi_{j}∥}^{2}-\sum_{j=0}^{N}{∥s_{j}∥}^{2}-\frac{1}{\gamma^{2}}\sum_{j=0}^{N}e_{j}^{T}e_{j}}}\leq \varepsilon$$

wherein $x_{0}$ and $P_{0}$ are the initial state vector and the initial state covariance matrix and *ε* is a constant.

As was mentioned in [20], Equation (34) generate an ellipsoid set of the estimated state, whose boundary is restrained by *ε*. Therefore, the estimation of the robust $H_{\infty }$ filter is under this constraint, as well. However, with uncertain parameters, the quadratic function is irreversible directly with the Gramian matrix of the noise error in the Krein space.

Therefore, based on Theorem 1 in [20], vectors in the Hilbert space are indicated by the vectors that have the same meanings in the Krein space. Then, the objective quadratic Equation (34) can be rewritten as:

(35) $$\;J_{\infty }\left(x_{0},u,v\right)={x_{0}}^{T}{P_{0}}^{-1}x_{0}+\sum_{j=0}^{N}\left\{u_{j}^{T}Q_{j}^{-1}u_{j}+v_{j}^{T}v_{j}+\xi_{j}^{T}\xi_{j}-s_{j}^{T}s_{j}-\frac{1}{\gamma^{2}}e_{j}^{T}e_{j}\right\}$$

Since that zero vector will not change the quadratic value, the following zero polynomial is introduced:

(36) $$\;\begin{array}{c} 0=\sum_{j=0}^{N}\left\{2\xi_{j}^{T}D_{j}^{T}R_{j}^{-1}D_{j}\xi_{j}\right.+v_{j}^{T}R_{j}^{-1}D_{j}\xi_{j}+\xi_{j}^{T}D_{j}^{T}R_{j}^{-1}v_{j}\\ \;\;\;\;\;\;\;\;\;\;\;\;\;\;\;-\left.\xi_{j}^{T}D_{j}^{T}R_{j}^{-1}\left(v_{j}+D_{j}\xi_{j}\right)-\left({v_{j}+D_{j}\xi_{j}}^{T}\right)R_{j}^{-1}D_{j}\xi_{j}\right\} \end{array}$$

Substituting the above equation into Equation (35), we can get:

(37) $$\begin{array}{ll} J_{\infty }\left(x_{0},u,v\right)= & {x_{0}}^{T}{P_{0}}^{-1}x_{0}+\sum_{j=0}^{N}\left\{u_{j}^{T}Q_{j}^{-1}u_{j}-s_{j}^{T}s_{j}-\frac{1}{\gamma^{2}}e_{j}^{T}e_{j}\right.\\ & \;\left.+{\left(\begin{array}{c} \xi_{j}\\ v_{j}+D_{j}\xi_{j} \end{array}\right)}^{T}\left(\begin{array}{cc} I+D_{j}^{T}R_{j}^{-1}D_{j} & -D_{j}^{T}R_{j}^{-1}\\ -R_{j}^{-1}D_{j} & R_{j}^{-1} \end{array}\right)\left(\begin{array}{c} \xi_{j}\\ v_{j}+D_{j}\xi_{j} \end{array}\right)\right\} \end{array}$$

After matrix inversion, Equation (37) is equivalent to:

(38) $$\;J_{\infty }\left(x_{0},u,v\right)={x_{0}}^{T}{P_{0}}^{-1}x_{0}+\sum_{j=0}^{N}{\left(\begin{array}{c} u_{j}\\ D_{j}v_{j} \end{array}\right)}^{T}\left(\begin{array}{cc} Q_{j} & S_{j}D_{j}^{T}\\ D_{j}S_{j}^{T} & D_{j}R_{j}D_{j}^{T} \end{array}\right)\left(\begin{array}{c} u_{j}\\ D_{j}v_{j} \end{array}\right)$$

Based on the Equation (7) in the Krein space, the coordinate transformation is as follows:

(39) $$\;\left(\begin{array}{c} x_{0}\\ u\\ \Omega v \end{array}\right)=\left(\begin{array}{ccc} I & 0 & 0\\ 0 & I & 0\\ -\Theta & -\Upsilon & I \end{array}\right)\left(\begin{array}{c} x_{0}\\ u\\ y \end{array}\right)$$

Then, the vector form of Equation (38) is:

(40) $$\;J_{\infty }\left(x_{0},u,v\right)={\left(\begin{array}{c} x_{0}\\ u\\ \Omega v \end{array}\right)}^{T}{\left(\begin{array}{ccc} P_{0} & 0 & 0\\ 0 & Q & S\Omega^{T}\\ 0 & \Omega S^{T} & \Omega R\Omega^{T} \end{array}\right)}^{-1}\left(\begin{array}{c} x_{0}\\ u\\ \Omega v \end{array}\right)$$

Therefore, the quadratic function can be transformed as the following equation:

(41) $$\begin{array}{c} J_{\infty }\left(x_{0},u,y\right)={\left(\begin{array}{c} x_{0}\\ u\\ y \end{array}\right)}^{T}{\left\{\left(\begin{array}{ccc} I & 0 & 0\\ 0 & I & 0\\ \Theta & \Upsilon & I \end{array}\right)\left(\begin{array}{ccc} P_{0} & 0 & 0\\ 0 & Q & S\Omega^{T}\\ 0 & \Omega S^{T} & \Omega R\Omega^{T} \end{array}\right){\left(\begin{array}{ccc} I & 0 & 0\\ 0 & I & 0\\ \Theta & \Upsilon & I \end{array}\right)}^{T}\right\}}^{-1}\left(\begin{array}{c} x_{0}\\ u\\ y \end{array}\right) \end{array}$$

Obviously, the weighted matrix of the quadratic function and the one of the error covariance matrix in the Krein space are just inverse. Therefore, we can design recursive steps of the $H_{\infty }$ filter in the Krein space.

Considering a state model in the Krein space represented as Equation (1), the recursive steps of the $H_{\infty }$ filter are summarized as follows:

(42) $$\;x_{j\left|j-1\right.}=A_{j-1}x_{j-1\left|j-1\right.}$$

(43) $$\;\begin{array}{c} P_{j\left|j\right.}=A_{j-1}P_{j-1\left|j-1\right.}{A_{j-1}}^{T}+B_{j-1}{B_{j-1}}^{T}\\ \;\;\;\;\;\;\;\;\;\;-A_{j-1}P_{j-1\left|j-1\right.}\left[\begin{array}{cc} C_{j}^{T} & L_{j}^{T} \end{array}\right]{\left(R_{e}\right)}^{-1}\left[\begin{array}{c} C_{j}\\ L_{j} \end{array}\right]P_{j-1\left|j-1\right.}{A_{j-1}}^{T} \end{array}$$

(44) $$\;{\left(R_{e}\right)}^{-1}=\left[\begin{array}{cc} I & 0\\ 0 & -\gamma^{2}I \end{array}\right]+\left[\begin{array}{c} C_{j}\\ L_{j} \end{array}\right]P_{j-1\left|j-1\right.}\left[\begin{array}{cc} C_{j}^{T} & L_{j}^{T} \end{array}\right]$$

(45) $$\;K_{j}=P_{j\left|j\right.}C_{j}^{T}{\left(I+C_{j}P_{j\left|j\right.}C_{j}^{T}\right)}^{-1}$$

(46) $$\;x_{j\left|j\right.}=x_{j\left|j-1\right.}+K_{j}\left(y_{j}-C_{j}x_{j\left|j-1\right.}\right)$$

Compared to the KF, the proposed filter is much more robust. Actually, when γ→∞, the $H_{\infty }$ filter is just the KF exactly. Additionally, the less *γ* is, the stronger the robustness of the system. Using the above $H_{\infty }$ filter, the uncertainty of the system noise can be eliminated availably. Hence, taking void measurement into account, we introduce a new parameter *α* into the presented robust $H_{\infty }$ filter. Assume that the coefficient of the void measurement is indicated as $\alpha \left(1\geq \alpha \geq 0\right)$:

when α=1, the CNS measurement is constantly available;

when α<1, the CNS measurement is lost.

That means, the smaller *α* is, the more the measurement is invalid. Therefore, the proposed novel robust $H_{\infty }$ filter in the Krein space can be rewritten as the following reliability:

The predicted state is still as Equation (42), while the predicted measurement is:

(47) $$\;{\hat{y}}_{j}=\alpha C_{j}x_{j\left|j-1\right.}$$

To derive the filtering steps conveniently, we define some intermediate variables:

(48) $$\;C_{j}^{\infty }=\left[\begin{array}{c} \alpha C_{j}\\ L_{k} \end{array}\right]$$

(49) $$\;{\left(R_{e}^{\infty }\right)}^{-1}=C_{j}^{\infty }P_{j-1\left|j-1\right.}{\left(C_{j}^{\infty }\right)}^{T}+\left[\begin{array}{cc} \alpha \left(1-\alpha \right)C_{j}C_{j}^{T} & 0\\ 0 & -\gamma^{2}I \end{array}\right]$$

(50) $$\;K_{e}^{\infty }=P_{j-1\left|j-1\right.}\left(C_{j}^{\infty }\right){\left(R_{e}^{\infty }\right)}^{-1}$$

(51) $$\;P_{j}^{\infty }=\left(I-K_{e}^{\infty }C_{j}^{\infty }\right)P_{j-1\left|j-1\right.}$$

Therefore, the estimated covariance matrix:

(52) $$\;P_{j\left|j\right.}=A_{j-1}P_{j}^{\infty }{A_{j-1}}^{T}+A_{j-1}{A_{j-1}}^{T}$$

The filtering gain is:

(53) $$\;K_{j}=P_{j}^{\infty }C_{j}^{T}\left(I+\alpha \left(1-\alpha \right)IC_{j}C_{j}^{T}\right)$$

The estimated state:

(54) $$\;x_{j\left|j\right.}=x_{j\left|j-1\right.}+K_{j}\left(y_{j}-{\hat{y}}_{j}\right)$$

In the SINS/CNS integrated system, the measurement of the CNS is invalid occasionally for the environmental disturbance. To solve various uncertainty problems, the robust $H_{\infty }$ filter introduces the $H_{\infty }$ norm, which is the robust design parameter. In this filter, the noise and the uncertainty are regarded as the limited energy random signal. Then, the filter can be designed based on the objective quadratic that the $H_{\infty }$ norm of the transfer function from the system interference to the estimated error is less than a given positive threshold.

## 4. Simulations and Experiments

To verify and estimate the performance of the proposed robust filter, simulations and experiments are performed in this section.

### 4.1. Simulations and Analysis

First of all, numerical simulations have been done. Suppose that the initial parameters of a marine vehicle are given and shown as Table 1:

According to Section 2.2, the state vector is composed of the position errors δL and δλ, the velocity errors $\delta V_{E},\delta V_{N}$, the misalignment angle errors $\phi_{E},\phi_{N},\phi_{U}$, the gyroscope constant drifts $\varepsilon_{x},\varepsilon_{y},\varepsilon_{z}$ and the accelerometer constant biases $\nabla_{x},\nabla_{y}$. The measurement vector is the attitude error between the SINS and CNS. On the basis of Equations (16)–(26), the model of the SINS/CNS integrated system can be expressed clearly.

In order to estimate the performance of the proposed filter, the normal KF is used as the reference filter. The initial state vector is:

(55) $$\begin{array}{c} x_{0}={\left[\begin{array}{cccccccccccc} 0\;m & 0\;m & 0\;\text{m/s} & 0\;\text{m/s} & 20^{\prime } & 20^{\prime } & 20^{\prime } & 0.01^{\circ }/h & 0.01^{\circ }/h & 0.01^{\circ }/h & 10^{-4}g_{0} & 10^{-4}g_{0} \end{array}\right]}^{T} \end{array}$$

Additionally, the corresponding covariance matrix is:

(56) P0=diag1010ReRe2,1010ReRe2,0.12,0.12,20′2,20′2,20′2,0.01∘/h2,0.01∘/h2,0.01∘/h2,10-4g02,10-4g02

wherein Re indicates the Earth’s radius, Re= 6,378,393.0 m.

**Figure 2:**
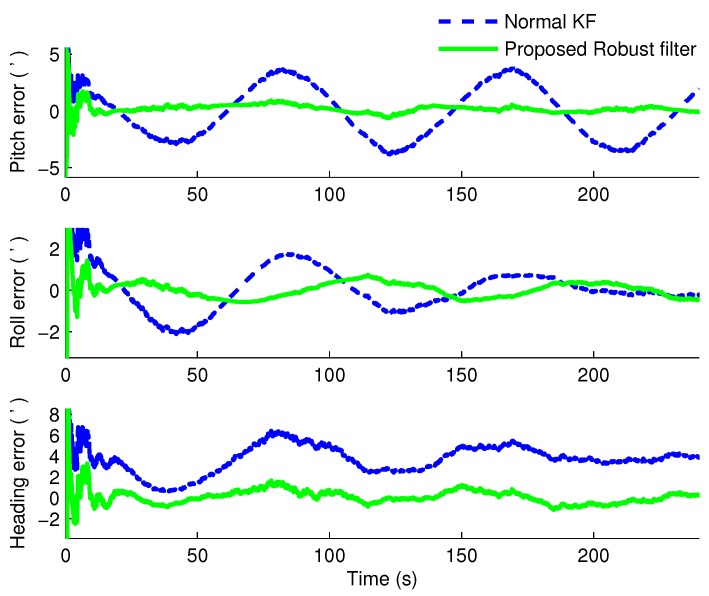
The estimated errors of the misalignment angles when α=0.75.

**Figure 3:**
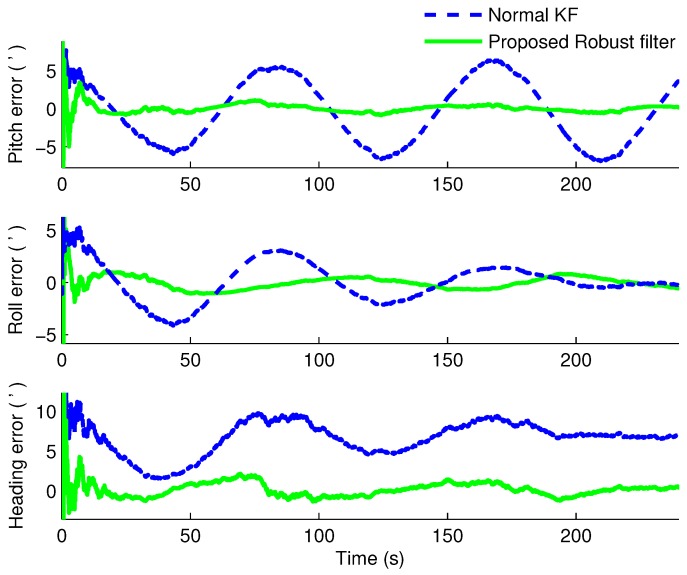
The estimated errors of the misalignment angles when α=0.5.

Under the same simulation conditions, the proposed robust $H_{\infty }$ filter and the normal KF were applied to estimate the states of the SINS/CNS integrated navigation system. We compared the errors of misalignment angles in different conditions when α=0.75 and α=0.5, respectively. The estimated results are shown in Figure 2 and Figure 3, respectively. To compare the running times of these two filters, Monte Carlo simulations are carried out 20-times on the same computer equipped with an Intel Core 2 T6570, 2.1-GHz processor and 3 GB RAM under Windows XP. Additionally, the means of the running times are compared in Table 2.

From Figure 2 and Figure 3, it is obvious that when α=0.75, the heading errors with the KF and proposed robust $H_{\infty }$ filter are 4’ and −0.3’, respectively; when α=0.5, the heading error with the robust $H_{\infty }$ filter is merely 0.4’, while the one with KF is about 7’. The average running times of normal Kalman filter are 0.7146 s and 0.6972 s, while the average times of the proposed robust filter are 0.9691 s and 0.9457 s when α=0.75 and α=0.5, respectively. Therefore, we can know that obviously when the CNS measurement is lost occasionally, the estimated accuracy of the normal KF is decreased severely from 4’ to 7’. In addition, the more measurements are lost, the worse the estimated results are. However, under these two conditions, utilizing the proposed robust $H_{\infty }$ filter, the misalignment angles not only can be estimated availably, but also have better convergence rates and stability than the KF method at a cost of few computations. Therefore, the robustness and superiority of the proposed $H_{\infty }$ filter can be verified.

### 4.2. Experiments and Analysis

**Figure 4:**
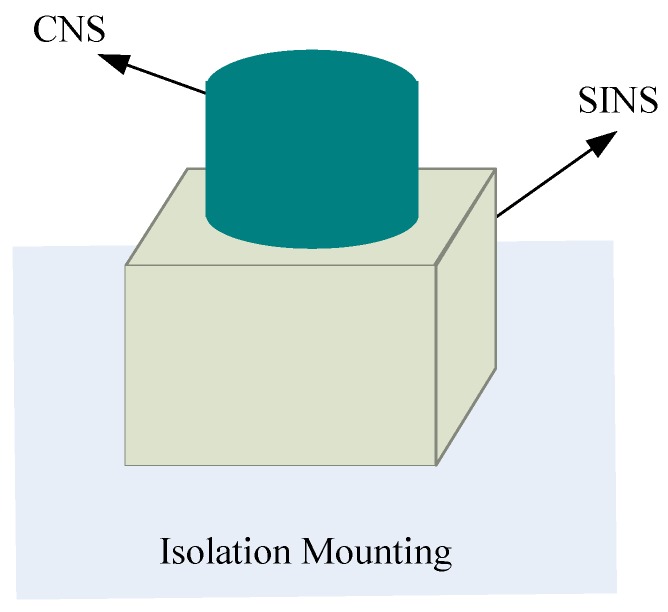
Schematic of the experimental setup.

**Figure 5:**
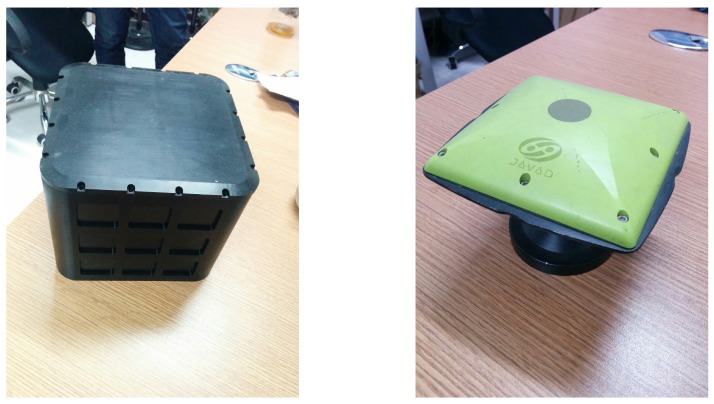
The IMU (**left**) and GPS (**right**) used in the experiments.

To further validate the performance of the proposed robust $H_{\infty }$ filter based on the Krein space theory, some experiments are carried out, as well. In these experiments, the SINS and star sensor are fixed on the ship’s deck together. The schematic of experimental setup and sensors are shown in Figure 4 and Figure 5, respectively. In the SINS, the inertial measurement unit (IMU) is composed of the accelerometer and the gyroscope, which was developed by our lab and shown as the left picture in Figure 5. The performance parameters of the SINS and the star sensor are detailed in Table 3.

During the experiment, the lens of the star sensor is covered discontinuously to simulate the invalid state of the star Sensor from about 22 h to 24 h. In addition, the GPS is also used as the reference information, shown as the right picture in Figure 5. Therefore, in the experiments, the SINS/GPS integrated system can be used as the standard to estimate the accuracy of the SINS/CNS integrated system.

**Figure 6:**
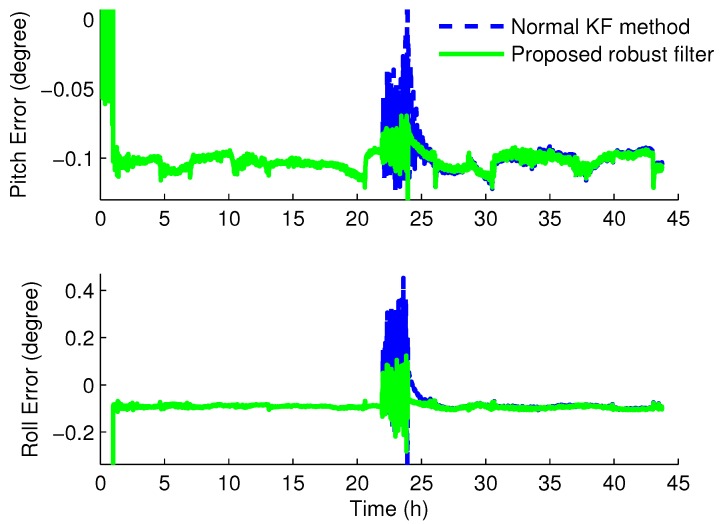
The horizontal angle errors with the normal Kalman filter method and the proposed robust $H_{\infty }$ filter. The blue dash line indicates the horizontal errors by utilizing the normal Kalman filter method; The green solid line indicates the horizontal errors by utilizing the novel method proposed in this article.

**Figure 7:**
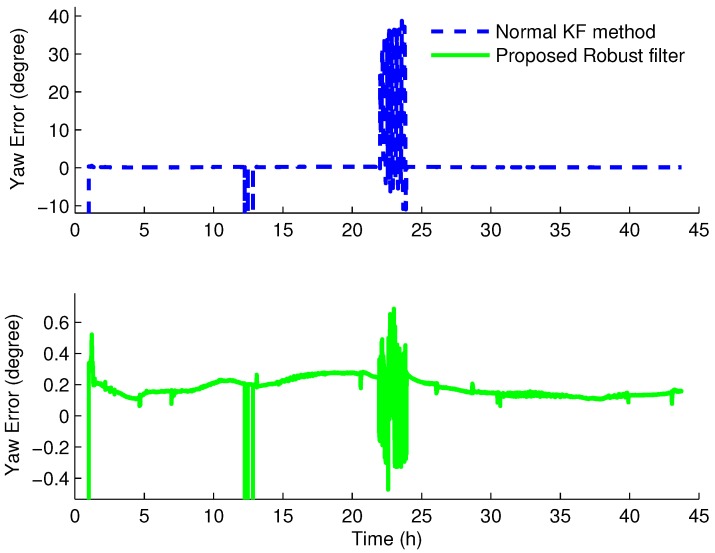
The azimuth angle errors with the normal Kalman filter method and the proposed robust $H_{\infty }$ filter. The blue dash line indicates the azimuth errors by utilizing the normal Kalman filter method; The green solid line indicates the azimuth errors by utilizing the novel method proposed in this article.

Figure 6 and Figure 7 give the experiment results of the SINS/CNS integrated system with the normal KF and robust $H_{\infty }$ filter based on the Krein space theory, respectively.

From the experiment results, we can see that the angle errors are vibrational when the star sensor is covered from about 22 h to 24 h. For the pitch error, the amplitude values are about $0.14^{\circ }$ and $0.11^{\circ }$ with the normal KF and with the proposed robust filter, respectively, while the values of the roll error are $0.44^{\circ }$ and $0.21^{\circ }$. Compared to the normal KF method, the horizontal and azimuth angle errors of the SINS/CNS integrated system are a bit smaller with the new robust filter based on the $H_{\infty }$ filter and the Krein space theory. Regarding the yaw error, the maximal errors of the normal KF and the proposed robust filter have an extremely great distance. With the traditional KF method, the maximal are $38.76^{\circ }$, as the one with the proposed robust filter algorithm is nearly $0.69^{\circ }$. Therefore, with the proposed robust filter, the attitude errors can be decreased dramatically when the measurements of the integrated system are uncertain or lost. Furthermore, the robustness of this novel robust filter is also verified.

## 5. Conclusions

In order to solve the uncertainty problem of the SINS/CNS integrated navigation system caused by the missing measurements, a novel robust $H_{\infty }$ filter based on the Krein space theory was proposed in this manuscript. Firstly, the system model of the SINS/CNS integrated navigation system was established, and then, a novel robust filter taking the uncertainty problem into account was proposed. Then, the superiority of the Krein space was described in principle, and the derivational process of the novel robust $H_{\infty }$ filter was presented in detail. Numerical simulations and experiments were carried out to verify the new robust $H_{\infty }$ filter. The results proved the advantages of the presented robust $H_{\infty }$ filter on the basis of the Krein space theory, which can improve the navigation accuracy of the integrated navigation system availably when the CNS measurements are lost. Therefore, the feasibility and the superiority of this new robust filter were verified. However, the system model of the SINS/CNS integrated system was assumed as a linear model, which is clearly unrealistic in practical applications. Therefore, our future work will focus on the nonlinear robust filter, which is suitable for practical systems.

## Figures and Tables

**Table 1:** Simulation parameters.

Parameters	Values
initial latitude	$L=40.2631049^{\circ }$
initial longitude	$\lambda =120.886482^{\circ }$
initial velocity	$v_{x}=v_{y}=0$
gravity acceleration	$g_{0}=9.7805$ m/s${}^{2}$
initial misalignment angles	$\phi_{x}=\phi_{y}=\phi_{z}=20^{\prime }$
constant drifts of the gyroscopes	$\varepsilon_{x}=\varepsilon_{y}=\varepsilon_{z}=0.01^{\circ }$/h
random noise of the gyroscopes	$W_{gx}=W_{gy}=W_{gz}=0.05^{\circ }$/h
constant biases of the accelerometers	$\nabla_{x}=\nabla_{y}=10^{-4}g_{0}$
random noise of the accelerometer	$W_{ax}=W_{ay}=5*10^{-5}g_{0}$
sampling frequency	98 Hz

**Table 2:** Comparisons of the running times.

	Running Time (s)	
	Kalman Filter	Proposed Filter
α=0.5	0.6972	0.9457
α=0.75	0.7146	0.9691

**Table 3:** Main parameters of the SINS and star sensor.

Sensors	Parameters	Values
Gyro	Dynamic range	$\pm 100^{\circ }$/s
	Bias stability	$\leq 0.01^{\circ }$/h
	Random walk	$\leq 0.005^{\circ }/\sqrt{h}$
	Scale factor stability	≤20 ppm
Accelerometer	Dynamic range	±4 g
	Bias stability	$\leq 10^{-4}$ g
	Random walk	$\leq 5*10^{-5}$ g
	Scale factor stability	≤20 ppm
Star Sensor	Field of view	$24^{\circ }$
	Attitude accuracy	$5^{\prime \prime }$
	Data update frequency	20 Hz
